# Alteration of cartilage surface collagen fibers differs locally after immobilization of knee joints in rats

**DOI:** 10.1111/joa.12290

**Published:** 2015-05-04

**Authors:** Momoko Nagai, Tomoki Aoyama, Akira Ito, Junichi Tajino, Hirotaka Iijima, Shoki Yamaguchi, Xiangkai Zhang, Hiroshi Kuroki

**Affiliations:** 1 Department of Motor Function Analysis, Human Health Sciences, Graduate School of Medicine, Kyoto UniversityKyoto, Japan; 2 Department of Development and Rehabilitation of Motor Function, Human Health Sciences, Graduate School of Medicine, Kyoto UniversityKyoto, Japan

**Keywords:** cartilage, collagen fiber, immobilization, rat model, scanning electron microscopy

## Abstract

The purpose of this study was to examine the ultrastructural changes of surface cartilage collagen fibers, which differ by region and the length of the experimental period in an immobilization model of rat. Male Wistar rats were randomly divided into histological or macroscopic and ultrastructural assessment groups. The left knees of all the animals were surgically immobilized by external fixation for 1, 2, 4, 8 or 16 weeks (*n *= 5/time point). Sagittal histological sections of the medial mid-condylar region of the knee were obtained and assessed in four specific regions (contact and peripheral regions of the femur and tibia) and two zones (superficial and deep). To semi-quantify the staining intensity of the collagen fibers in the cartilage, picrosirius red staining was used. The cartilage surface changes of all the assessed regions were investigated by scanning electron microscopy (SEM). From histological and SEM observations, the fibrillation and irregular changes of the cartilage surface were more severe in the peripheral region than in the contact region. Interestingly, at 16 weeks post-immobilization, we observed non-fibrous structures at both the contact and peripheral regions. The collagen fiber staining intensity decreased in the contact region compared with the peripheral region. In conclusion, the alteration of surface collagen fiber ultrastructure and collagen staining intensity differed by the specific cartilage regions after immobilization. These results demonstrate that the progressive degeneration of cartilage is region specific, and depends on the length of the immobilization period.

## Introduction

Joint immobilization has various influences on joint structure, articular cartilage degeneration as disuse atrophy (Jurvelin et al. 1985; Setton et al. 1997), and concomitant alterations in other joint disorders such as pain or limitation in the range of motion (Vanwanseele et al. 2002; Hagiwara et al. 2009). Understanding the degeneration or atrophy of articular cartilage is necessary to develop strategies to prevent and treat joint disease, such as osteoarthritis or secondary degeneration after contracture (Moriyama et al. 2008; Hagiwara et al. 2009).

The articular cartilage consists of chondrocytes embedded in a substantial amount of extracellular matrix (ECM), which is primarily composed of type II collagen and the proteoglycan (PG) aggrecan (Schachar et al. 1999; Moger et al. 2009; Leong et al. 2010). Additionally, collagen fibers are important for the cartilage skeleton (Vanwanseele et al. 2002; Moger et al. 2009). Previous reports have shown that joint immobilization increases collagen synthesis, maintains or elevates collagen content (Tammi et al. 1983, 1988; Saamanen et al. 1987), decreases type II collagen (Hagiwara et al. 2010) as well as PG content (Haapala et al. 1996, 1999), and upregulates the expression of matrix metalloproteinases (Ando et al. 2009; Leong et al. 2010), which have been considered the main enzymes responsible for the degeneration of collagens in articular cartilage (Nagase & Kashiwagi, 2003). The effect of immobilization can alter collagen fibers and the matrix by differences in the method of immobilization, the loading conditions, the duration of immobilization and the assessment region (Jurvelin et al. 1986; Moriyama et al. 2008). Previous reports (Jortikka et al. 1997; Haapala et al. 1999) showed that the characterization of cartilage PG was well-described zonally and regionally in a canine immobilization model, but there are few comparable reports in rats.

The differences in cartilage change according to the assessment region, which has been demonstrated histologically in a rat immobilization model. The assessment regions were mainly divided into two regions as follows: non-contact and contact regions (O'Connor, 1997; Trudel et al. 2005; Moriyama et al. 2008). The contact region is located where the articular cartilages of two bones are in contact with one another, and previous reports showed similar findings such as the degeneration of chondrocytes and cartilage thinning in this region (Trudel et al. 2005; Hagiwara et al. 2009). Additionally, some reports evaluated histological and elasticity alterations with additional regions, including the peripheral to the contact region (the peripheral region) along with the above-mentioned two regions after immobilization (Ando et al. 2009; Hagiwara et al. 2009, 2010). In addition, Hagiwara et al. (2009) reported that immobilization leads to the deterioration of histological scoring and softening of the cartilage in both the contact and peripheral regions. However, the ultrastructural changes of cartilage dissimilarity among these assessment regions remain unclear. Macroscopic and microscopic examinations of the articular cartilage after immobilization revealed surface irregularities and softening (Jurvelin et al. 1985; Trudel et al. 2003; Hagiwara et al. 2009). The cartilage surface, which directly faces the joint cavity, is at risk for cartilage and chondrocyte degeneration when the cartilage is cleaved off from the surface by horizontal fissuring (Clark & Simonian, 1997). Previous reports showed that ultrastructural degenerative alterations of the cartilage surface after immobilization occurred in the cartilage of various bones of the knee joint, including the femur (Jozsa et al. 1987; Clark, 1990), tibia (Helminen et al. 1983; Clark, 1990) and patella (Jurvelin et al. 1983, 1985; Clark, 1990; Hong & Henderson, 1996; Clark & Simonian, 1997). These reports examined the entire articular surface of one bone but not the separate regions. Consequently, little is known about the ultrastructural alterations of specific cartilage regions, including the contact and peripheral regions.

The purpose of this study was to examine the ultrastructural changes of cartilage collagen fibers, which differ by region. Therefore, we focused on the contact and peripheral regions, which differ based on the length of the experimental period (i.e. short- or long-term) in an immobilization rat model to identify differences in specific cartilage regions.

## Materials and methods

### Sample preparation and surgical procedure

The experimental design for this study was approved by the College Animal Research Committee of Kyoto University (Permission number: 12597). We used a total of 50 male Wistar rats, 8 weeks old, weighing 178–217 g that were randomly allocated in groups of 25 for the histology analysis and 25 for the macroscopic observation and scanning electron microscopy (SEM) analysis at the following five time points: 1, 2, 4, 8 and 16 weeks after surgical immobilization. The left hind limb of each experimental animal was immobilized with an external fixator consisting of wire and resin (immobilized). Under sodium Nembutal anesthesia and sterile conditions, Kirschner wires were screwed into the femur and the tibia, and fixed with wire and resin to maintain knee flexion of approximately 140 ± 5 ° by the previously described method (Nagai et al. 2014). The right knee joint was subjected to sham surgery and was freely movable postoperatively (control). All the experimental animals had their left knee joint immobilized, which did not damage the joint. All the animals were housed in groups of two or three in plastic cages in an environmentally controlled room, and fed food and water *ad libitum*.

### Histological observations

At the end of the immobilization period, the animals were killed under anesthesia with sodium Nembutal by exsanguination. After the wire and resin were removed from the joint, a microscopic analysis was performed. The knees were removed, fixed with 4% paraformaldehyde at 4 °C overnight, and thereafter decalcified in 10% ethylene diamimine-tetetraacetic acid at 4 °C. The decalcified samples were embedded in paraffin. Using standard procedures, 6-μm sections were stained with hematoxylin-eosin (H-E) to observe surface and cell changes; they were also stained with picrosirius red to observe the cartilage collagen fibers. The samples from all the time points and the controls were stained with picrosirius red simultaneously. The sections were observed under a microscope.

#### Assessment regions

**Fig 1:**
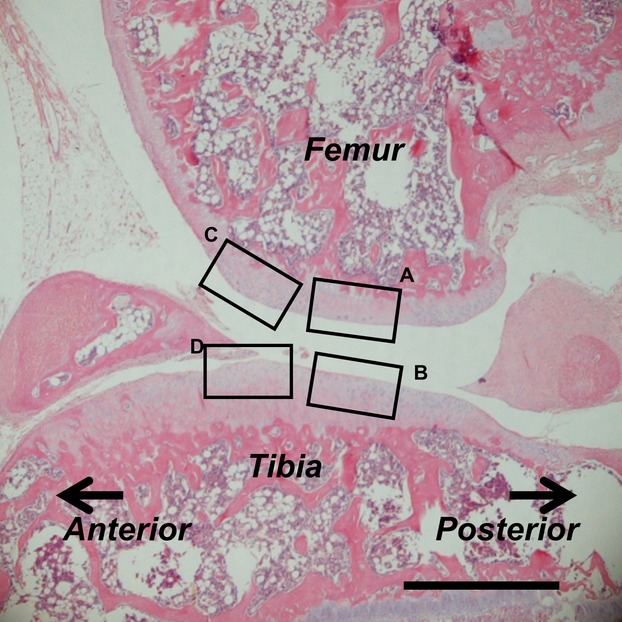
Assessment regions in the histological imaging of a standard sagittal section of the knee (stained with H-E at 8 weeks post-immobilization). (A) Femur contact region; FC. (B) Tibia contact region; TC. (C) Femur peripheral region; FP. (D) Tibia peripheral region; TP. Scale bar: 500 μm.

After immobilization, the medial side is more affected by degeneration and thickness of the cartilage than the lateral compartment (Haapala et al. 1999, 2000) or in osteoarthritis (Ledingham et al. 1993). Therefore, we only assessed the medial side of the knee joint. We observed histological alterations at the medial mid-condylar region of the knee in the sagittal plane of the articular cartilage. Four regions were evaluated (Fig.1). The femur contact region (FC; Fig.1A) is the dominant weight-bearing region of the femur and is located at apposed regions when the knee is flexed to 140 ° (consistent with the angle of immobilization). The tibia contact region (TC; Fig.1B) is the weight-bearing surface of the tibia that is distributed across the surface of the tibia when the knee is flexed to 140 °. The peripheral region includes both the femur peripheral region (FP; Fig.1C) and the tibia peripheral region (TP; Fig.1D), which are 0.6 ± 0.1 mm away from the center of each respective contact region. The peripheral regions were defined by measuring the distance from the center of the contact regions to the area at which a convex shape formed around the center of the FC or TC regions.

#### Measurement of picrosirius red staining

To semi-quantify the collagen content in the cartilage, one way to visualize collagen fibers in histological sections is by staining with picrosirius red (Beesley et al. 1992; Schmitz et al. 2010). The staining intensity of picrosirius red at each of the four regions was measured separately in the superficial and deep zones of cartilage. Rectangles that were 50 μm deep and 400 μm long were superimposed over the histological sections. The superficial zone of cartilage was defined as the region from the cartilage surface down to a depth of 50 μm. The deep zone of cartilage was defined as the region from the tidemark up to a depth of 50 μm. The images from each sample were measured using the ImageJ program (US National Institutes of Health). TIFF images were taken at a magnification of  ×200 under microscopy using the standard protocols; then, the TIFF images were converted to eight-bit gray scale images, followed by inverting black and white values and subtracting the background. The average intensity of each zone was calculated. The intensity ranged from 0 to 255 and represented the intensity of collagen fibers from low to high.

### Macroscopic observations

After the rats were killed under anesthesia, the specimens for the macroscopic and SEM observations were fixed in 2% glutaraldehyde in 4% paraformaldehyde at 4 °C overnight. Based on our macroscopic observations, we extracted the femur and tibia from the fixed knee joints. These procedures were performed to prevent dehydration and damage from affecting the cartilage surface. The extracted femur and tibia were observed frontally and laterally using a Keyence VB-7010 camera (Keyence, Osaka, Japan).

### SEM

After the macroscopic observations, the specimens were cleaved with a scalpel and washed in 0.1 m phosphate buffer, and a second fixation step was performed with 1% osmic acid. The specimens were dehydrated in a graded series of ethanol, transferred into tert-butyl alcohol, and freeze-dried at −20 °C. The dried specimens were mounted on stages, coated with platinum/palladium and observed using a HITACHI S-4700 electron microscope (Hitachi High Technologies, Tokyo, Japan). We observed the alterations of the cartilage surface collagen in the FC, TC, FP and TP regions.

### Statistical analysis

All the data are shown as the mean ± standard deviation (SD). The software program JMP 11 (SAS Institute, Cary, NC, USA) was used for the statistical analysis. Differences in the staining intensity between the immobilized and control rats at each time point were assessed using the Mann–Whitney's *U*-test. In all cases, *P *< 0.05 was considered significant.

## Results

### Macroscopic observations

**Fig 2:**
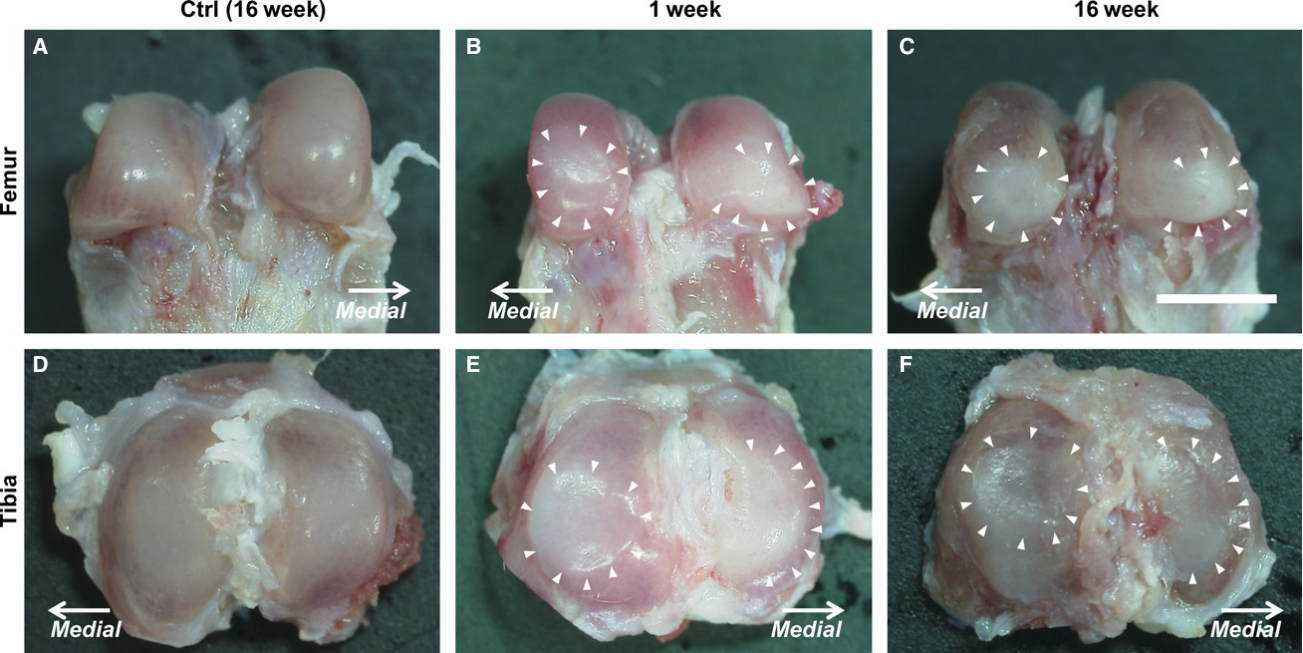
Macroscopic images of the femur and tibia. (A, D) In the control group, an intact cartilage surface can be observed at 16 weeks post-operation. The images show (A–C) the femur and (D–F) the tibia. In the immobilized group (B, E), the 1-week post-immobilization images show that the surface of the contact region was concave in shape and encircled by protruding cartilage that formed a convex shape (arrowheads show the edge of the convex shape). The surface of the convex shape changed in color from red to white. (C, F) At 16 weeks post-immobilization, more severe alterations than that of the previous experimental periods can be observed. The macrographs have the same magnification: ×25; scale bar: 3.00 mm.

There was no apparent macroscopic degeneration of the cartilage in any of the specimens of the control group throughout the experimental period (Fig.2A,D). However, in the immobilized group, the surfaces of the contact regions in the femur and tibia were concave and encircled by protruding cartilage that was convex in shape (Fig.2B,C,E,F; arrowheads show the edge of the convex shape). The surface color of the convex shape in the femur changed from red to white (Fig.2B,C). These color and shape changes became more severe throughout the experimental period, and were the most severe in the immobilized group at 16 weeks (Fig.2C,F).

### Histological observations

**Fig 3:**
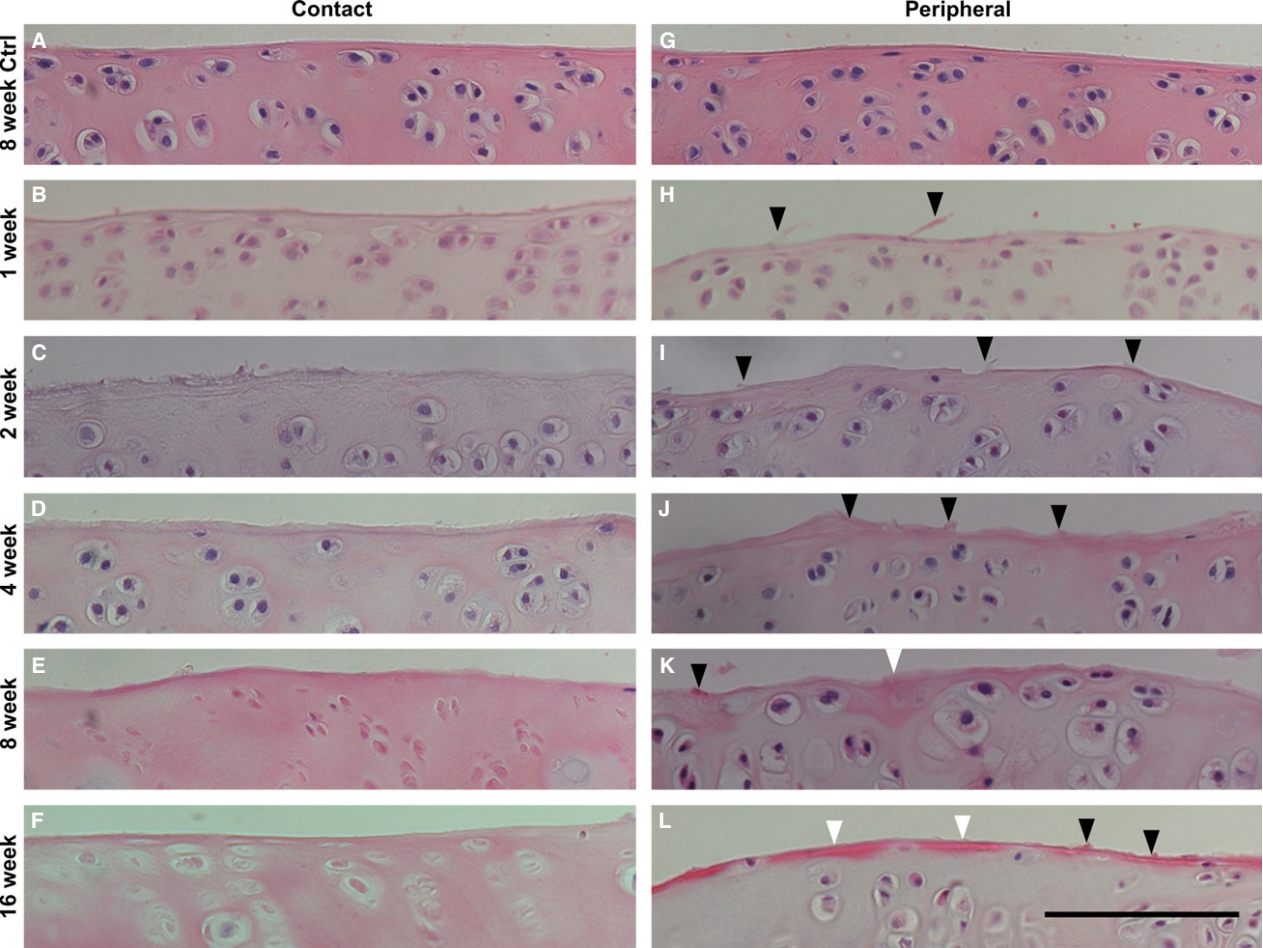
Histological images of the superficial zone of the contact and peripheral regions in the femur were stained with H-E. (A, G) In the control group, no apparent histological degeneration of the cartilage and surface was observed at 8 weeks. In the immobilized groups, the images show (B, H) 1 week, (C, I) 2 weeks, (D, J) 4 weeks, (E, K) 8 weeks and (F, L) 16 weeks post-immobilization. (B–F) The contact region in the immobilized groups does not show remarkable fibrillation of the cartilage surface. Hypertrophied cells and degenerative changes of the chondrocytes were observed, particularly at 8 and 16 weeks post-immobilization. (H–L) The peripheral region in the immobilized group shows changes in fibrillation and irregularity (black arrowheads) and alterations in the staining density (white arrowheads) of the cartilage surface. Hypertrophy of the chondrocytes was observed throughout the experimental period. The micrographs have the same magnification: ×400; scale bar: 100 μm.

The histological changes in the cartilage surface were similar to the changes in the femur and tibia throughout the experimental period. The H-E staining showed no apparent histological degeneration in any of the control group specimens throughout the experimental period (Fig.3A,G). In the immobilized group, at the contact region, hypertrophic chondrocytes were observed throughout the experimental period, and degenerative changes of the chondrocytes particularly in the chondrocytic lacunae with pyknotic or absent nuclei were observed predominantly at 8 and 16 weeks (Fig.3B–F). However, the fibrillation and irregular changes of the cartilage surface were more severe in the peripheral region than in the contact region at the same time point (Fig.3H–L, black arrowheads). The remarkable fibrillation that was identified at 2–4 weeks post-immobilization was not observed at 8 and 16 weeks, but the changes in the densely stained surface were observed (Fig.3K,L, white arrowheads). Moreover, hypertrophic chondrocytes were observed in the peripheral region throughout the experimental period (Fig.3H–L). The results were uniform for all the specimens that belonged to the same group.

### Measurement of picrosirius red staining

#### Contact region

**Fig 4:**
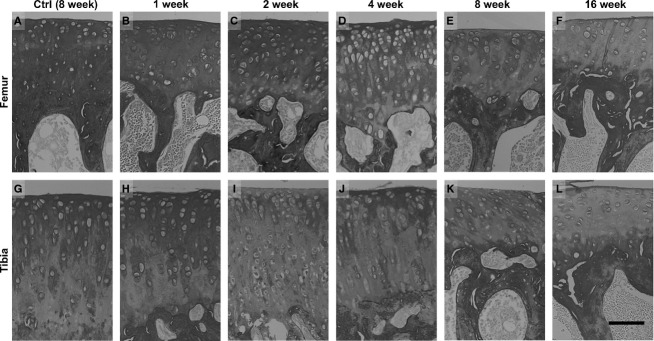
Histological imaging of the contact regions at the femur and tibia cartilage, which were stained with picrosirius red converted to gray scale. (A, G) In the control group, high staining intensity can be observed at 8 weeks post-operation. In the immobilized groups, the images show (B, H) 1 week, (C, I) 2 weeks, (D, J) 4 weeks, (E, K) 8 weeks and (F, L) 16 weeks post-immobilization. (B–F) The femur in the immobilized group has a reduced staining intensity with the prolongation of the experimental period. (H–L) The tibia in the immobilized group has a reduced staining intensity after 2 weeks post-immobilization, except for the image at 4 weeks. The micrographs have the same magnification: ×200; scale bar: 100 μm.

**Fig 5:**
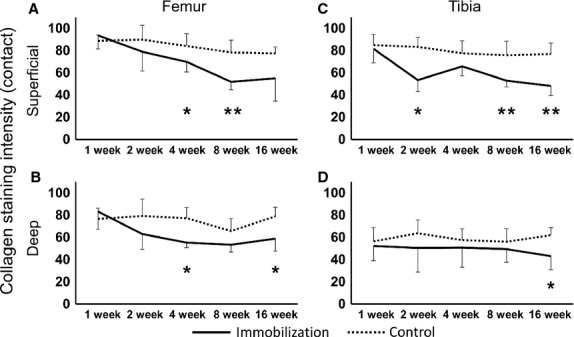
Collagen fibers intensity staining at the contact region of the femur and tibia. (A) The superficial zone of the femur in the immobilized knees showed a significant decrease in intensity at 4 and 8 weeks post-immobilization. (B) The deep zone of the femur in the immobilized knees showed a significant decrease at 4 and 16 weeks post-immobilization. (C) The superficial zone of the tibia in the immobilized knees showed a significant decrease in intensity at 2, 8 and 16 weeks post-immobilization. (D) The deep zone of the tibia in the immobilized knees showed a significant decrease in intensity at 16 weeks post-immobilization. Values are presented as mean ± SD; control vs. immobilized at the same time point (*P < 0.05, **P < 0.01).

The intensity of the picrosirius red staining of the control group remained constant throughout the experimental period (Fig.4A,G). However, in the immobilized group, the intensity significantly decreased at the superficial zone in the femur at 4 and 8 weeks (Figs4D,E and 5A: *P < 0.05, **P < 0.01), and in the tibia at 2, 8 and 16 weeks (Figs4I,K,L and 5C: *P < 0.05, **P < 0.01) compared with that of the control. There were no significant differences in the femurs, although the intensity was decreased at 8 and 16 weeks compared with 2 and 4 weeks post-immobilization (Fig.4E,F). In the deep zone, the staining intensity showed significant decreases in the femurs at 4 and 16 weeks, and in the tibias at 16 weeks compared with that of the control (Figs4D,F,L and5B,D: *P < 0.05).

#### Peripheral region

**Fig 6:**
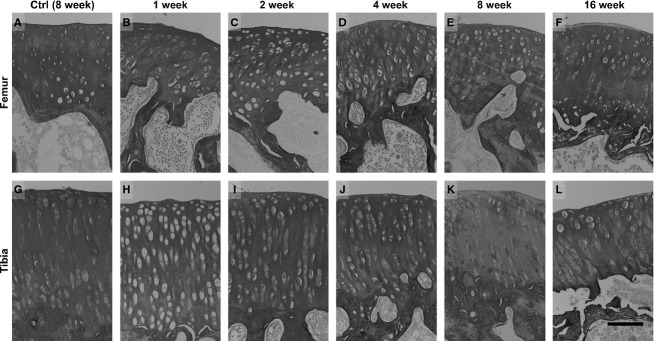
Histological imaging of the peripheral regions at the femur and tibia cartilage, which were stained with picrosirius red converted to gray scale. (A, G) In the control group, high staining intensity was observed at 8 weeks post-operation. In the immobilized groups, the images show (B, H) 1 week, (C, I) 2 weeks, (D, J) 4 weeks, (E, K) 8 weeks and (F, L) 16 weeks post-immobilization. (B–F) The femur and (H–L) tibia in the immobilized group retained their staining intensity throughout the experimental period, except in both the superficial and deep zones in the tibia at 8 weeks post-immobilization. The micrographs have the same magnification: ×200; scale bar: 100 μm.

**Fig 7:**
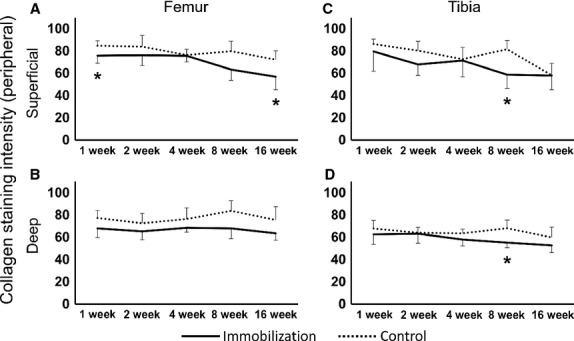
Collagen fibers intensity staining at the peripheral region of the femur and tibia. (A) The superficial zone showed a significant decrease in intensity at 1 and 16 weeks post-immobilization. (B) The deep zone of the femur in the immobilized knees showed no significant differences in intensity throughout the experimental period. (C) The superficial zone and (D) the deep zone of the tibia in the immobilized knees: there were no significant differences throughout the experimental period except at 8 weeks post-immobilization. Values are presented as mean ± SD; control vs. immobilized at the same time point (*P < 0.05).

The intensity of the picrosirius red staining of the control groups remained constant throughout the experimental period (Fig.6A,G). However, in the immobilized group, the staining intensity of the peripheral region differed from that of the contact region. The intensity was almost constant throughout the experimental period. However, at the superficial zone, the intensity was significantly decreased in the femur at 1 and 16 weeks (Figs6B,F and 7A: *P < 0.05), and in the tibia at 8 weeks (Figs6K and 7C: *P < 0.05) compared with that of the control. At the deep zone, the intensity significantly decreased at only 8 weeks in the tibia (Figs6K and 7B,D: *P < 0.05).

### SEM

**Fig 8:**
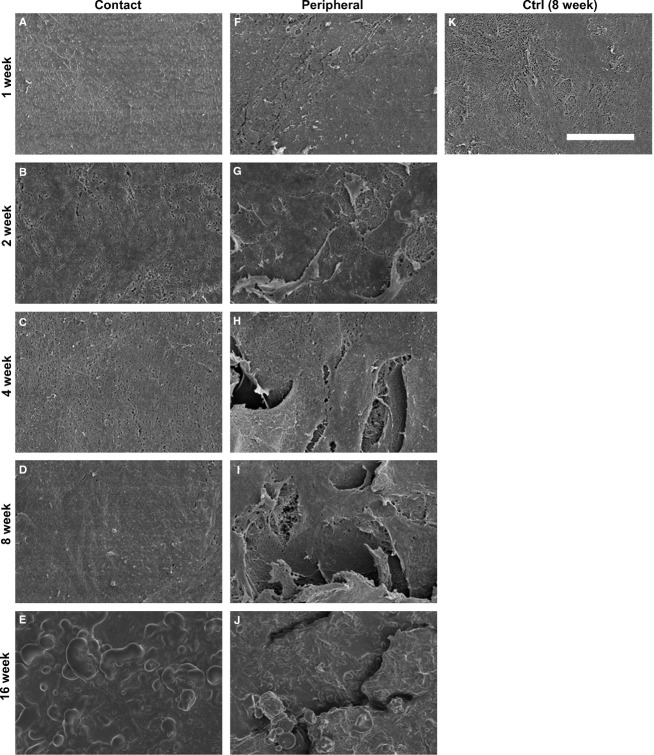
SEM images of the surface at the contact and peripheral regions of the femur. The images show (A, F) 1 week, (B, G) 2 weeks, (C, H) 4 weeks, (D, I) 8 weeks and (E, J) 16 weeks post-immobilization. (K) The contact region in the control group at 8 weeks post-operation shows collagen fibrous structures on the cartilage surface, and these structures were observed in all the assessment regions throughout the experimental period in the control group. (A–C) At the contact region in the immobilized group, the mashed fiber network was observed at 1, 2 and 4 weeks post-immobilization. (D) A slightly uneven but not leafy and knobby surface was observed at 8 weeks post-immobilization. (E) A hillocky surface comprised of non-fibrous structures was observed at 16 weeks post-immobilization. (F, G) At the peripheral region in the immobilized groups, an uneven, leafy and split surface was observed at 1 and 2 weeks post-immobilization. (H, I) The larger leafy surface and split was observed at 4 and 8 weeks post-immobilization. (J) The slightly rough and knobby surface comprised of non-fibrous structures was observed at 16 weeks post-immobilization. The SEMs have the same magnification: ×1500; scale bar: 20 μm.

The collagen fiber changes in the cartilage surface were similar in the femur and tibia throughout the entire experimental period. In the contact region, the surface was even and smooth, and collagen fibrillar structures were observed in the control group throughout the experimental period (Fig.8K). However, in the contact region, a mashed fiber network and cross-linking were observed after 1–4 weeks in the immobilized group (Fig.8A–C). Furthermore, slightly uneven but not leafy and knobby surfaces were observed at 8 weeks (Fig.8D), and hillocky surfaces comprised of non-fibrous structures were observed at 16 weeks (Fig.8E). In the peripheral region, the even and smooth surface was comprised of collagen fibrillar structures, which was similar to the contact region; this was observed in the control group throughout the experimental period. However, in the immobilized group, uneven and slightly rough surfaces were observed at 1 week (Fig.8F). The leafy and slightly split surfaces were observed at 2 weeks (Fig.8G). The leafy surfaces were larger than those at 2 weeks; moreover, splitting of the surface beneath the collagen fiber was observed at 4 and 8 weeks (Fig.8H,I). The rough and knobby surfaces were comprised of non-fibrous structures that covered this region at 16 weeks. Furthermore, we did not observe a leafy split, which can see fiber structure beneath the split (Fig.8J). The results were uniform for all the specimens that belonged to the same group.

## Discussion

We investigated the ultrastructural changes of the cartilage surface that differ by specific region (i.e. contact or peripheral regions). To the best of our knowledge, this is the first report addressing the differences between regions with regard to ultrastructural changes of surface collagen fibers after immobilization over a long-term experimental period.

Previous studies showed that significant alterations of collagen fibers in the entire cartilage surface of one bone were observed within 1 week post-immobilization, and the leafy, knobby or split structure of the cartilage surface was observed throughout 8 weeks immobilized period in a rabbit patella by SEM (Jurvelin et al. 1983, 1985). Moreover, one report showed that surface irregularities appeared across the entire cartilage surface after 2 weeks of immobilization and rapidly progressed after 8 weeks, which was determined by evaluating histological images of the tibia (Trudel et al. 2003). The present study revealed the fibrillation and irregular changes of the cartilage surface in the immobilized group throughout the experimental period by histological and ultrastructural observations (Figs3 and 8). Our 1–8-week post-immobilization results were consistent with previously reported results. In addition, at 16 weeks post-immobilization, non-fibrous structures were observed at both the contact and peripheral regions (Fig.8E,J). Many reports have shown the alteration of surface cartilage after long-term immobilization in the patella (Jurvelin et al. 1983, 1985; Hong & Henderson, 1996). However, in the tibia and femur, previous reports only reported a period of up to 8 weeks post-immobilization (Helminen et al. 1983; Jozsa et al. 1987). Thus, this is the first report of the ultrastructural alterations of collagen fiber in the surface of the femur and tibia cartilage after a long-term experimental period (i.e. 16 weeks post-immobilization).

Our results show that the alterations of collagen fibers in the cartilage surface differed by specific cartilage region (i.e. contact or peripheral regions). The surface at the contact region was concave in shape (Fig.2B,C,E,F) and consisted of a cross-linking fiber network (Fig.8A–C). However, the surface of the peripheral region was convex in shape (Fig.2B,C,E,F), and the leafy surface, which was more fibrillated than that of the contact region, was determined by histology (Fig.3H–L) and ultrastructure (Fig.8F–J). Based on the SEM observations, the leafy and split surfaces were observed at the peripheral region in the immobilized groups and became heavier throughout the immobilized period (Fig.8F–J). Regarding the differences between the regions, we suggested that there was a difference in mechanical stress. At the contact region, where the articular cartilage of two bones is in direct contact, compressive stress was loaded (Trudel et al. 2005). However, at the peripheral region, the articular cartilage may contact the meniscus. We could consider that not only compressive stress but also shear stress was loaded at the peripheral region because other structures of the joint, such as the meniscus or joint capsule, were not fixed whereas the bones were fixed. The type of load on the cartilage (i.e. compressive or shear stress) influenced chondrocyte metabolism (Smith et al. 2004; Ko et al. 2013). Cyclic compression causes cartilage degeneration (Milentijevic et al. 2003; Ko et al. 2013), and the application of shear stress increases the release of proinflammatory mediators and decreases aggrecan and type II collagen expression (Smith et al. 2004). Under mechanical loads, the water content is forced out of the matrix (Milentijevic et al. 2003), which facilitates the deformation of collagen in the upper zone of the cartilage (Kaab et al. 1998, 2000). The differences in the alterations of the surface cartilage by region may be affected by the differences in mechanical stress.

In this study, non-fibrous structures were observed by SEM at both the contact and peripheral regions 16 weeks post-immobilization, which could be caused by the effect of the synovial fluid on the cartilage surface. After immobilization, previous reports showed that a decreased amount of fluid (Enneking & Horowitz, 1972; Michelsson & Hunneyball, 1984) and decreased levels of TIMP (tissue inhibitors of metalloproteinase) were present in the synovial fluid (Haapala et al. 2001). The breakdown of homeostasis in the joint space led to fibril changes on the surface. However, because we did not investigate synovial fluid, we cannot confirm this possibility. The present results show that the alterations of the cartilage surface occurred at both the contact and peripheral regions, and the alteration pattern differed by specific region, indicating that further alterations of the cartilage surface that differ by specific regions may occur with a prolonged immobilization period or other new stresses such as re-load or re-joint movements.

The present study revealed that the alteration of collagen fiber intensity was different between the contact and peripheral regions. When we considered the changes for both superficial and deep zones, at the contact regions, the collagen fiber staining intensity significantly decreased compared with that of the control at the early phases of post-immobilization: after 2 weeks post-immobilization in the tibia, except for the deep zone; and after 4 weeks post-immobilization in the femur (Figs4 and 5). At the peripheral regions, the specific fluctuation of intensity confirmed which contact regions were not observed in both superficial and deep zones throughout the experimental period (Figs6 and 7). The superficial zone of cartilage comprises 10–20% of the cartilage thickness and has the ability to maintain fluid load support for compressive loading (Vanwanseele et al. 2002; Moger et al. 2009). The decreased collagen staining intensity at the superficial zone of the TC region was observed at an earlier phase in the immobilized period compared with that of the femur (Fig.5A,C). The deep zone of cartilage is defined as the area above the calcified cartilage, which comprises 30% of the cartilage thickness, and it consists of radially oriented fiber bundles that enter the calcified zone to form an interlocking network that anchors the tissue to the bony substrate (Vanwanseele et al. 2002). The deceased collagen staining intensity at the deep zone of the FC region was observed at an earlier phase in the immobilized period compared with that of the tibia (Fig.5B,D).

A depression in the collagen staining intensity indicates a change in the collagen diameter and packing density (Beesley et al. 1992; Schmitz et al. 2010). Our results on the alterations of collagen staining intensity showed a change in the collagen fiber structure after immobilization. Based on the SEM observations, we assessed the alteration of collagen fibers in detail using cross-sections of the TC region, which was where the collagen fiber stainability changed the most, to compare the different regions (Fig. S1). We confirmed the ultrastructural changes of collagen fibers at an early phase of the immobilization period. The collagen fibers appeared to be sparse at the transitional zone, and the fiber orientation was random, not vertical, at the radial zone. These results suggested that changes in the collagen fibers occurred ultrastructually in both the superficial and deep zones at an early phase of the immobilization period.

Although the exact origin is not known, two hypothetical mechanisms might explain the ultrastructural collagen changes in articular cartilage after immobilization. The first hypothesis pertains to the effect of the degeneration of chondrocytes to collagen fiber and cartilage matrix. Many reports have shown that chondrocyte degeneration or apoptosis occur at the contact region in cartilage after rigid immobilization (Haapala et al. 1999; Trudel et al. 2005; Hagiwara et al. 2009). Immobilization causes reduced PG content (Haapala et al. 1996, 1999), decreased collagen cross-linking, decreased tissue content of uronic acid (Haapala et al. 1996) and suppression of PG synthesis (Palmoski et al. 1980; Behrens et al. 1989). Knee cartilage atrophy, which occurs after immobilization, is accompanied by these changes in the chondrocytes surrounding the ECM. To our knowledge, the decreased collagen fiber staining intensity indicates alterations in the ECM content of the cartilage after immobilization. The second hypothesis pertains to the effect of mechanical properties. Previous biomechanical studies have shown that mechanical loading can either stimulate or inhibit biosynthetic activity (Sah et al. 1989; Kaab et al. 1998), and can influence the arrangement and orientation of collagen fibers (Moger et al. 2009). Previous reports also showed that excessive compression causes cartilage degeneration, and the structure of articular cartilage collagen exhibits zone-specific deformation that is dependent on the type of load, such as cyclic or static (Kaab et al. 1998, 2000). These alterations of chondrocytes and the ECM may reflect the staining intensities of the collagen fibers that were observed in this study. Furthermore, Ando et al. (2011) reported hypertrophy of the chondrocytes in the contact and peripheral regions through increased mechanical stress by rigid immobilization that was irreversible after remobilization. Alterations of surface cartilage collagen fibers and the collagen staining intensity, which was observed in this report, may indicate a similar irreversible alteration as that observed by Ando et al. (2011).

However, this study has several potential limitations. First, we used immature, 8-week-old rats. Previous reports showed that collagen content doubles (Williamson et al. 2001) and growth affects the collagen structure (Julkunen et al. 2010) during the maturation process. In this study, it is possible that the results were affected by the inhibition of normal cartilage fiber growth. Second, we only focused on collagen fiber in the cartilage. Therefore, alterations of other components, such as large aggregating and non-aggregating PGs that are also components of the articular cartilage matrix, are unknown. Third, the assessment area was the medial side of the knee joint; thus, changes of the lateral side of the knee joint remain unexplained. Fourth, we used a small sample size for the statistical analysis of the staining intensity. A higher number of samples at the each time point is desirable to have more power for the statistical analysis.

## Concluding remarks

The ultrastructure of surface collagen fibers changed remarkably at the peripheral region compared with the contact region with prolongation of the immobilized period of knee joint articular cartilage. Additionally, the collagen fiber staining intensity significantly decreased at the contact region compared with the peripheral region after immobilization. Cartilage degeneration was shown to be region specific (i.e. contact region or peripheral region). We suggest that the progressive degeneration of cartilage in specific regions occurs with prolongation of the immobilization period.

## Supporting Information

**Fig S1.** Scanning electron microscopy images of cross-sections of the contact region of the tibia.

## References

[b1] Ando A, Hagiwara Y, Tsuchiya M (2009). Increased expression of metalloproteinase-8 and -13 on articular cartilage in a rat immobilized knee model. Tohoku J Exp Med.

[b2] Ando A, Suda H, Hagiwara Y (2011). Reversibility of immobilization-induced articular cartilage degeneration after remobilization in rat knee joints. Tohoku J Exp Med.

[b3] Beesley JE, Jessup E, Pettipher R (1992). Microbiochemical analysis of changes in proteoglycan and collagen in joint tissues during the development of antigen-induced arthritis in the rabbit. Matrix.

[b4] Behrens F, Kraft EL, Oegema TR (1989). Biochemical changes in articular cartilage after joint immobilization by casting or external fixation. J Orthop Res.

[b5] Clark JM (1990). The organisation of collagen fibrils in the superficial zones of articular cartilage. J Anat.

[b6] Clark JM, Simonian PT (1997). Scanning electron microscopy of “fibrillated” and “malacic” human articular cartilage: technical considerations. Microsc Res Tech.

[b7] Enneking WF, Horowitz M (1972). The intra-articular effects of immobilization on the human knee. J Bone Joint Surg Am.

[b8] Haapala J, Lammi MJ, Inkinen R (1996). Coordinated regulation of hyaluronan and aggrecan content in the articular cartilage of immobilized and exercised dogs. J Rheumatol.

[b9] Haapala J, Arokoski JP, Hyttinen MM (1999). Remobilization does not fully restore immobilization induced articular cartilage atrophy. Clin Orthop Relat Res.

[b10] Haapala J, Arokoski J, Pirttimaki J (2000). Incomplete restoration of immobilization induced softening of young beagle knee articular cartilage after 50-week remobilization. Int J Sports Med.

[b11] Haapala J, Arokoski JP, Ronkko S (2001). Decline after immobilisation and recovery after remobilisation of synovial fluid IL1, TIMP, and chondroitin sulphate levels in young beagle dogs. Ann Rheum Dis.

[b12] Hagiwara Y, Ando A, Chimoto E (2009). Changes of articular cartilage after immobilization in a rat knee contracture model. J Orthop Res.

[b13] Hagiwara Y, Ando A, Chimoto E (2010). Expression of collagen types I and II on articular cartilage in a rat knee contracture model. Connect Tissue Res.

[b14] Helminen HJ, Jurvelin J, Kuusela T (1983). Effects of immobilization for 6 weeks on rabbit knee articular surfaces as assessed by the semiquantitative stereomicroscopic method. Acta Anat (Basel).

[b15] Hong SP, Henderson CN (1996). Articular cartilage surface changes following immobilization of the rat knee joint. A semi quantitative scanning electron-microscopic study. Acta Anat (Basel).

[b16] Jortikka M, Inkinen R, Tammi M (1997). Immobilisation causes longlasting matrix changes both in the immobilised and contralateral joint cartilage. Ann Rheum Dis.

[b17] Jozsa L, Jarvinen M, Kannus P (1987). Fine structural changes in the articular cartilage of the rat's knee following short-term immobilisation in various positions: a scanning electron microscopical study. Int Orthop.

[b18] Julkunen P, Halmesmaki EP, Iivarinen J (2010). Effects of growth and exercise on composition, structural maturation and appearance of osteoarthritis in articular cartilage of hamsters. J Anat.

[b19] Jurvelin J, Kuusela T, Heikkila R (1983). Investigation of articular cartilage surface morphology with a semiquantitative scanning electron microscopic method. Acta Anat (Basel).

[b20] Jurvelin J, Helminen HJ, Lauritsalo S (1985). Influences of joint immobilization and running exercise on articular cartilage surfaces of young rabbits. A semiquantitative stereomicroscopic and scanning electron microscopic study. Acta Anat (Basel).

[b21] Jurvelin J, Kiviranta I, Tammi M (1986). Softening of canine articular cartilage after immobilization of the knee joint. Clin Orthop Relat Res.

[b22] Kaab MJ, Ito K, Clark JM (1998). Deformation of articular cartilage collagen structure under static and cyclic loading. J Orthop Res.

[b23] Kaab MJ, Ito K, Rahn B (2000). Effect of mechanical load on articular cartilage collagen structure: a scanning electron-microscopic study. Cells Tissues Organs.

[b24] Ko FC, Dragomir C, Plumb DA (2013). *In vivo* cyclic compression causes cartilage degeneration and subchondral bone changes in mouse tibiae. Arthritis Rheum.

[b25] Ledingham J, Regan M, Jones A (1993). Radiographic patterns and associations of osteoarthritis of the knee in patients referred to hospital. Ann Rheum Dis.

[b26] Leong DJ, Gu XI, Li Y (2010). Matrix metalloproteinase-3 in articular cartilage is upregulated by joint immobilization and suppressed by passive joint motion. Matrix Biol.

[b27] Michelsson JE, Hunneyball IM (1984). Inflammatory involvement in rabbit knee following immobilization and resulting in osteoarthritis. Scand J Rheumatol.

[b28] Milentijevic D, Helfet DL, Torzilli PA (2003). Influence of stress magnitude on water loss and chondrocyte viability in impacted articular cartilage. J Biomech Eng.

[b29] Moger CJ, Arkill KP, Barrett R (2009). Cartilage collagen matrix reorientation and displacement in response to surface loading. J Biomech Eng.

[b30] Moriyama H, Yoshimura O, Kawamata S (2008). Alteration in articular cartilage of rat knee joints after spinal cord injury. Osteoarthritis Cartilage.

[b31] Nagai M, Aoyama T, Ito A (2014). Contributions of biarticular myogenic components to the limitation of the range of motion after immobilization of rat knee joint. BMC Musculoskelet Disord.

[b32] Nagase H, Kashiwagi M (2003). Aggrecanases and cartilage matrix degradation. Arthritis Res Ther.

[b33] O'Connor KM (1997). Unweighting accelerates tidemark advancement in articular cartilage at the knee joint of rats. J Bone Miner Res.

[b34] Palmoski MJ, Colyer RA, Brandt KD (1980). Joint motion in the absence of normal loading does not maintain normal articular cartilage. Arthritis Rheum.

[b35] Saamanen AM, Tammi M, Kiviranta I (1987). Maturation of proteoglycan matrix in articular cartilage under increased and decreased joint loading. A study in young rabbits. Connect Tissue Res.

[b36] Sah RL, Kim YJ, Doong JY (1989). Biosynthetic response of cartilage explants to dynamic compression. J Orthop Res.

[b37] Schachar NS, Novak K, Muldrew K (1999). Articular cartilage joint surface reconstruction techniques. J Orthop Sci.

[b38] Schmitz N, Laverty S, Kraus VB (2010). Basic methods in histopathology of joint tissues. Osteoarthritis Cartilage.

[b39] Setton LA, Mow VC, Muller FJ (1997). Mechanical behavior and biochemical composition of canine knee cartilage following periods of joint disuse and disuse with remobilization. Osteoarthritis Cartilage.

[b40] Smith RL, Carter DR, Schurman DJ (2004). Pressure and shear differentially alter human articular chondrocyte metabolism: a review. Clin Orthop Relat Res.

[b41] Tammi M, Saamanen AM, Jauhiainen A (1983). Proteoglycan alterations in rabbit knee articular cartilage following physical exercise and immobilization. Connect Tissue Res.

[b42] Tammi M, Kiviranta I, Peltonen L (1988). Effects of joint loading on articular cartilage collagen metabolism: assay of procollagen prolyl 4-hydroxylase and galactosylhydroxylysyl glucosyltransferase. Connect Tissue Res.

[b43] Trudel G, Himori K, Goudreau L (2003). Measurement of articular cartilage surface irregularity in rat knee contracture. J Rheumatol.

[b44] Trudel G, Himori K, Uhthoff HK (2005). Contrasting alterations of apposed and unapposed articular cartilage during joint contracture formation. Arch Phys Med Rehabil.

[b45] Vanwanseele B, Lucchinetti E, Stussi E (2002). The effects of immobilization on the characteristics of articular cartilage: current concepts and future directions. Osteoarthritis Cartilage.

[b46] Williamson AK, Chen AC, Sah RL (2001). Compressive properties and function-composition relationships of developing bovine articular cartilage. J Orthop Res.

